# The application research of AI image recognition and processing technology in the early diagnosis of the COVID-19

**DOI:** 10.1186/s12880-022-00753-1

**Published:** 2022-02-17

**Authors:** Wenyu Chen, Ming Yao, Zhenyu Zhu, Yanbao Sun, Xiuping Han

**Affiliations:** 1 Department of Respiration, Affiliated Hospital of Jiaxing University, Jiaxing, China; 2 Department of Pain Medicine Center, Affiliated Hospital of Jiaxing University, Jiaxing, China; 3 Yangtze Delta Region Institute of Tsinghua University, Zhejiang, No. 705, Asia Pacific Road, Nanhu District, Jiaxing, 314006 Zhejiang China; 4 Radiology Department, Affiliated Hospital of Jiaxing University, No. 1882 Zhonghuan South Road, Jiaxing, 314000 China

**Keywords:** Artificial intelligence, COVID-19, Diagnosis, Computed tomography

## Abstract

**Background:**

This study intends to establish a combined prediction model that integrates the clinical symptoms,the lung lesion volume, and the radiomics features of patients with COVID-19, resulting in a new model to predict the severity of COVID-19.

**Methods:**

The clinical data of 386 patients with COVID-19 at several hospitals, as well as images of certain patients during their hospitalization, were collected retrospectively to create a database of patients with COVID-19 pneumonia. The contour of lungs and lesion locations may be retrieved from CT scans using a CT-image-based quantitative discrimination and trend analysis method for COVID-19 and the Mask R-CNN deep neural network model to create 3D data of lung lesions. The quantitative COVID-19 factors were then determined, on which the diagnosis of the development of the patients' symptoms could be established. Then, using an artificial neural network, a prediction model of the severity of COVID-19 was constructed by combining characteristic imaging features on CT slices with clinical factors. ANN neural network was used for training, and tenfold cross-validation was used to verify the prediction model. The diagnostic performance of this model is verified by the receiver operating characteristic (ROC) curve.

**Results:**

CT radiomics features extraction and analysis based on a deep neural network can detect COVID-19 patients with an 86% sensitivity and an 85% specificity. According to the ROC curve, the constructed severity prediction model indicates that the AUC of patients with severe COVID-19 is 0.761, with sensitivity and specificity of 79.1% and 73.1%, respectively.

**Conclusions:**

The combined prediction model for severe COVID-19 pneumonia, which is based on deep learning and integrates clinical aspects, pulmonary lesion volume, and radiomics features of patients, has a remarkable differential ability for predicting the course of disease in COVID-19 patients. This may assist in the early prevention of severe COVID-19 symptoms.

## Background

Pneumonia is a highly contagious disease caused by the severe acute respiratory syndrome coronavirus 2 (SARS-CoV-2) infection that emerged in December 2019 [1, 2]. At the beginning of the epidemic in China, of 1,099 laboratory-confirmed COVID-19 patients, 5.0% were admitted to intensive care units (ICU), 2.3% received invasive mechanical ventilation, and 1.4% died [3, 4]. COVID-19 represents a wide spectrum of clinical manifestations, including fever, cough, and fatigue, which may cause fatal acute respiratory distress syndromes [4]. COVID-19 has been proven to be infectious from person to person [5], and the World Health Organization (WHO) has declared COVID-19 a pandemic [6]. Therefore, the identification of risk factor parameters and the establishment of accurate prognostic prediction models are expected to improve clinical outcomes. Planning for early intervention and enhancing surveillance is critical in the event of a pandemic.

Currently, the sarS-COV-2 reverse transcription polymerase chain reaction (RT-PCR) is the preferred method for the detection of COVID-19 [7]. However, this method has the disadvantages of being a time-consuming and having a high false negative rate [8]. Computed tomography (CT) has a natural advantage in displaying lung lesions, and it is an important tool for the diagnosis, treatment and prognosis evaluation of lung diseases including pneumonia [9]. Chest CT images of the patients with COVID-19 pneumonia can provide detailed information related to pathology, as well as quantitative measurement of the size of the lesion and the severity of pulmonary involvement [2, 10, 11]. Recent research has also demonstrated that while RT-PCR is negative, chest CT can reveal lung abnormalities [12, 13]. Therefore, CT is a valuable auxiliary diagnostic tool for the early diagnosis and genotyping of patients with suspected COVID-19 pneumonia.

AI technology is a diagnostic assistance technology that has progressed rapidly in recent years, with impressive achievement in many medical domains [14–16]. As an AI method, deep learning has shown important clinical value in the use of CT images to assist in the analysis of lung diseases [17–19]. Thanks to powerful feature learning capabilities, deep learning can automatically detect features related to clinical results from CT images. Recent studies have shown [20] that using CT scanning to establish an AI system to detect COVID-19 can help radiologists and clinicians treat patients suspected of COVID-19. Gozes et al. (2020) used commercial software RADLogics Inc to detect pulmonary nodules and ground-glass opacities on 3D thoracic CT scans, and combined with 2D convolutional neural networks to segment the lung area and diagnose COVID-19. The test achieved an AUC of 0.996, sensitivity of 98.2%, and specificity of 92.2% on a dataset of 107 cases [21].

Critically ill patients with COVID-19 pneumonia have a significant fatality rate. 1.6% of active cases are in a severe or critical condition [22], and the mortality rate of critically ill patients is as high as 61.5% [23]. To reduce the rate of severe illness and mortality, it is critical to identify patients who are at risk of critical illness and are most likely to benefit from intensive care therapy as soon as possible. We can create an early warning model of severe COVID-19 using the Recurrent Neural Network (RNN) deep neural network and a comprehensive analysis of the thoracic CT radiomics and the patient's clinical characteristics. We expect to apply this model to generate early predictions regarding confirmed COVID-19 patients, allowing us to make more appropriate hierarchical management decisions, enhance patient prognosis, and reduce social medical costs.

## Methods

### Data source

According to the standards of COVID-19 Diagnosis and Treatment Protocol (Trial 7th Edition) [24], this research included 386 cases in Tianyou Hospital, which is Affiliated with Wuhan University of Science and Technology, the First Affiliated Hospital of Zhejiang University School of Medicine, and the First hospital of Jiaxing. Among the confirmed COVID-19 patients, 205 of them have CT image samples, and each patient took one or more CT images during the treatment. A total of 522 packets of CT image samplefrom COVID-19 patients and 95 packets of CT image of normal people were collected at the same time. The main clinical basic information of the patients was collected and sorted out, including the patient's basic demographic data, basic comorbidities, epidemiological histories, classification of the severity of the condition at admission, changes in the condition during treatment, symptoms during treatment, and laboratory examinations results etc. The control group consisted of samples from healthy patients who had not been infected with COVID-19 over the same time period.

### Patient diagnosis and clinical classification

The included patients met the following diagnostic criteria: high-throughput sequencing or RT-PCR of nasopharyngeal swab specimens were positive. According to the standards of the COVID-19 Diagnosis and Treatment Protocol (Trial 7th Edition), this research divides COVID-19 pneumonia into mild, moderate, and severe types. Since there are no pneumonia indications in the images of patients with mild symptoms, the study classified mild pneumonia as moderate pneumonia as well, in order to distinguish clinically diagnosed severe COVID-19 pneumonia from the other two types.

### CT image labeling and quality control

In order to train and evaluate our semantic segmentation framework, we manually segmented 100 CT slices manifesting COVID-19 features from 10 patients. Annotation was done through polygons. The segmentation labels were used to distinguish the relevant pathological features of COVID-19 pneumonia from other common pneumonia. The annotation included lung fields and five commonly seen lesion categories, including Compliance of Lung (CL), ground glass shadow, pulmonary fibrosis, interstitial thickening, and pleural effusion. Three senior radiologists with 15 to 25 years of expertise annotated and assessed the segmentation.

In order to analyze the CT images of patients, all images were selected for quality control by deleting any scans that were low-quality or unreadable. All images were subjected to a hierarchical grading system that included two levels of qualified grading professionals with good professional expertise who could verify and correct the image labels. Each image that was imputed into the database began with a label that matched to the patient's diagnostic results. This was an initial quality check performed by radiologists with 5 to 15 years of clinical practice experience who acted as first-level graders to exclude images with serious artifacts or with significantly reduced image resolution. Then they looked at the CT images to see whether there were any lung lesions.

### Model construction and verification

**Fig. 1:**
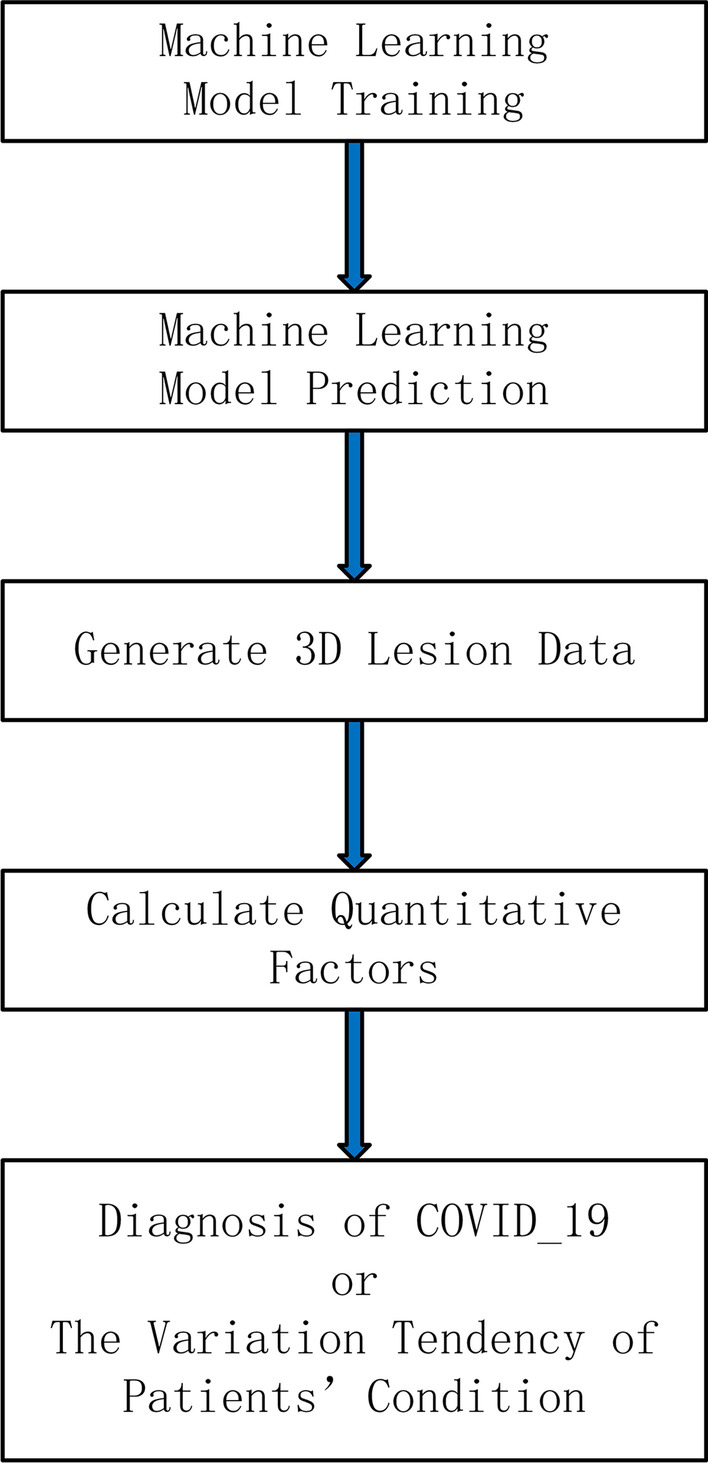
Flow chart of the quantitative discrimination and trend analysis algorithm of COVID-19 based on CT

**Fig. 2:**
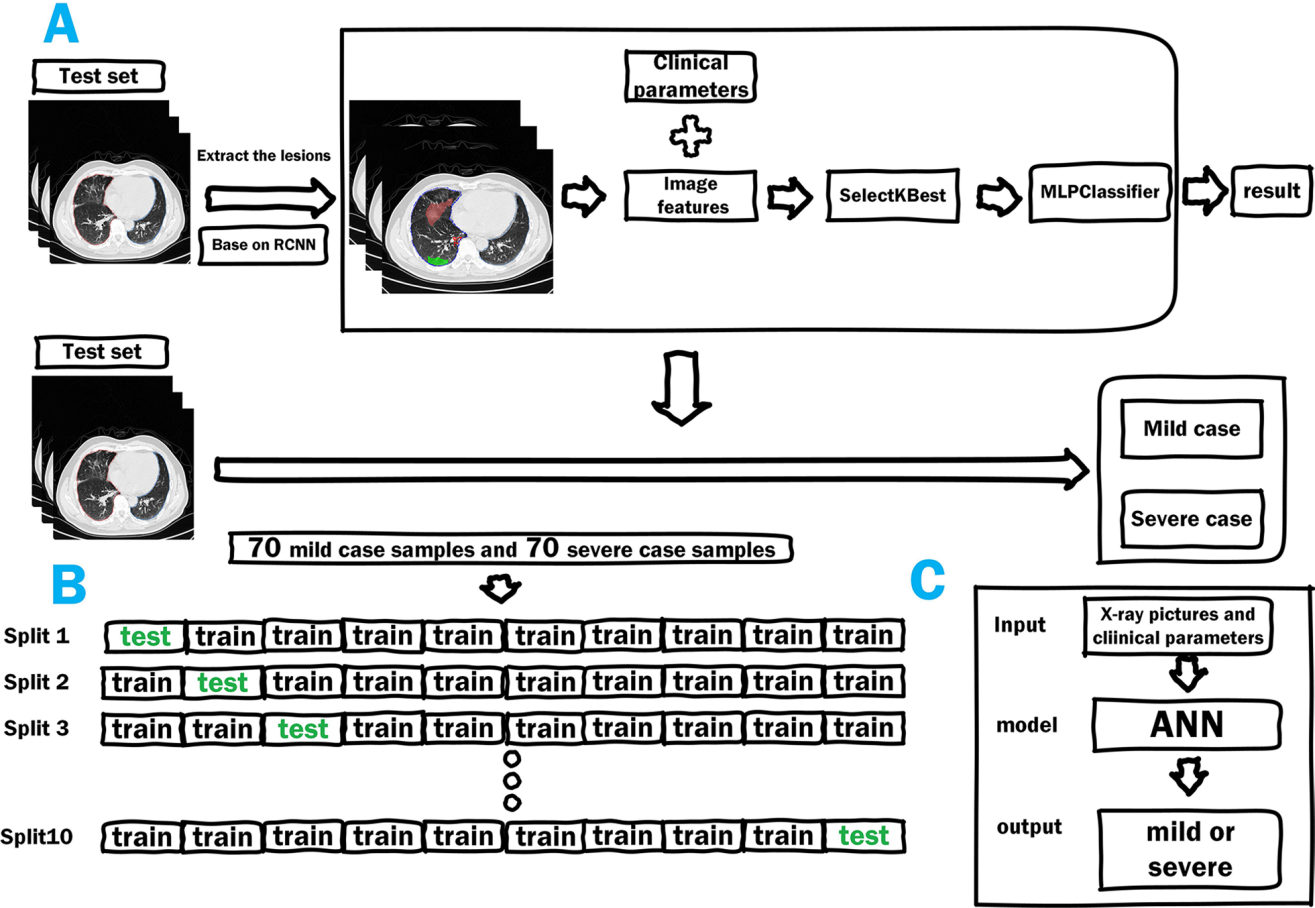
Design flow chart of the whole research

This research provides a CT-image-based COVID-19 pneumonia quantitative discrimination and trend analysis algorithm based on, through the Mask R-CNN deep neural network model [25], which extracts the contour of lungs and lesion locations from CT images to generate 3D lesion data and calculate COVID-19 pneumonia quantitative factors to determine whether the patient is infected by the pneumonia, and to determine the trend of patients’ condition (Fig. 1). Then, using CT imaging features and clinical parameters, an artificial neural network is used to create a prediction model for the severity of COVID-19. For training, an ANN is utilized, and the prediction model is validated using tenfold cross-validation (Fig. 2).

### Statistical analysis

The ROC and AUC were used to evaluate model performance. Sensitivity, specificity, and accuracy were determined by the selected operating point. The operating point between the low false-negative diagnosis rate (sensitivity) and the low positive diagnosis rate (1 − specificity) was set at different thresholds. The Pearson and Spearman correlation test of the Holm-Bonferroni Method was used for statistical analysis. The training, verification, and testing procedures of the deep learning model were carried out by using Pytorch (v.1.2.0). We used the Python scikit-learn library for data analysis [26] and used the Python matplotlib and seaborn libraries to draw graphics. We used the Python lightgbm and lifeline to predict prognosis. The measure value of sensitivity, specificity, and accuracy was also calculated by the Python scikit-learn library.

## Results

### Construction of a database of patients with COVID-19

**Fig. 3:**
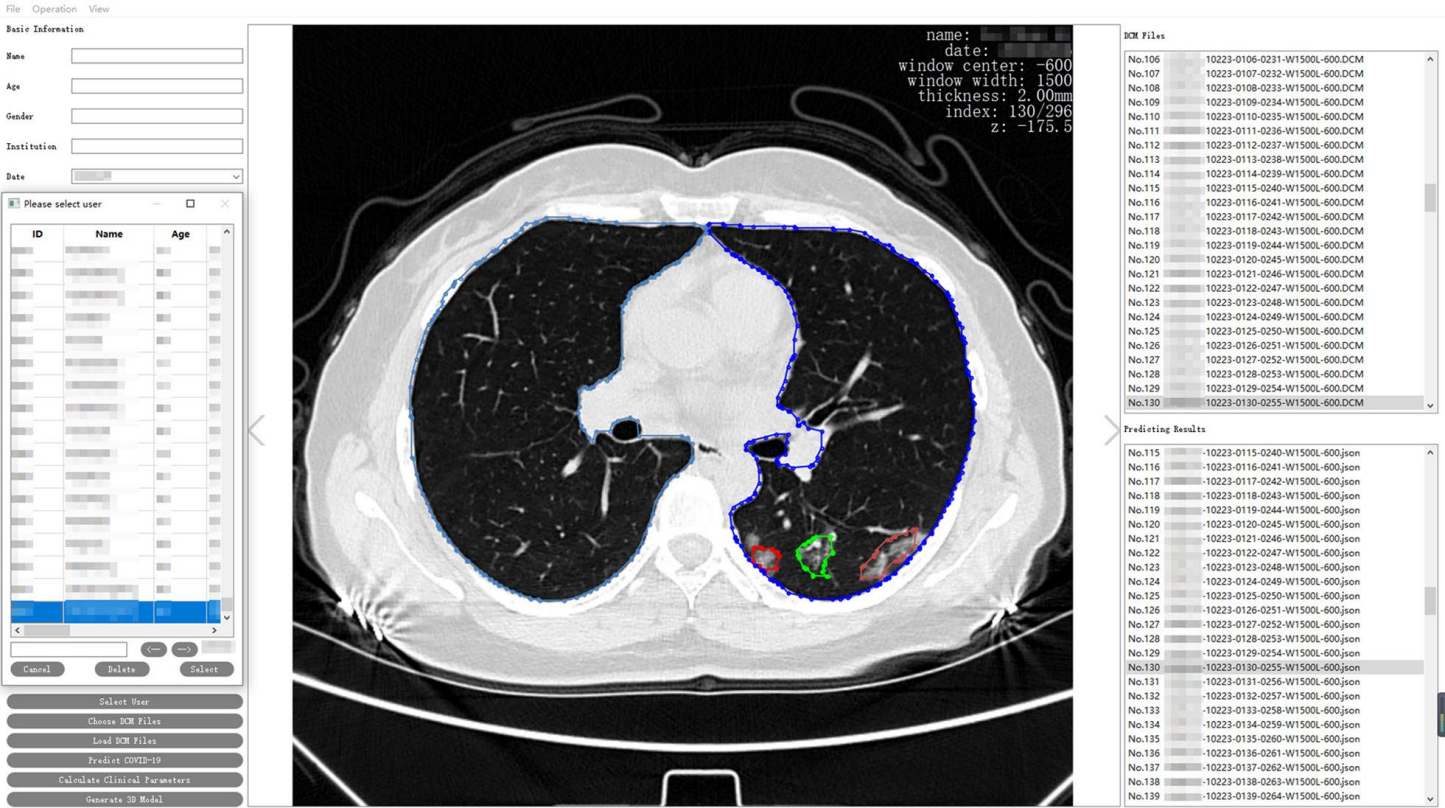
COVID-19 intelligent evaluation platform

Of 386 confirmed patients with COVID-19 included in this research, 205 had CT image specimens (Table 1); 207 (53.6%) were men and 179(46.4%) were women; the mean age of the patients was 57.3 years old; 362 (93.8%) had no previous smoking history; 293 (75.9%) had a history of underlining diseases, of which 45.3% had a history of two or more underlining diseases among which hypertension (123 cases) and diabetes (41 cases) being the most common; in the classification of severity of illness at the time of hospital admission, 45.6% of patients were mildly ill and 54.4% were critically or severely ill. During the treatment period, 47 patients who were mildly ill turned into critically ill patients. The data presented above suggested that the objects included in this research research can fully reflect the overall characteristics of the current COVID-19 patient population. The images of some patients during hospitalization were collected and analyzed, and these image files were archived and stored on the platform(Fig. 3).

**Table 1:** Patient’s clinical characteristics

Clinical characteristics information	Patient (n = 386), n(%)
Gender	
Male	207 (53.6%)
Female	179 (46.4%)
Age	
Average age(SD)	57.3(57.13 ± 15.77)
≤ 39	66 (17.1%)
40–49	48 (12.4%)
50–59	78 (20.2%)
60–69	109 (28.2%)
≥ 70	85 (22.1%)
Smoking history	
Yes	24 (6.2%)
No	362 (93.8%)
History of underlying disease	
0 underlying disease	93 (24.1%)
History of 1 underlying disease	118 (30.6%)
History of 2 or more underlying diseases	175 (45.3%)
Classification of illness at admission	
Mild	176 (45.6%)
Severe	205 (54.4%)

### The utilization of CT radiomics features extraction and analysis based on a deep neural network

Based on the characteristics of Mask R-CNN [25] transfer learning, only the above-mentioned 100 CT slice images containing lesion information were employed, with 80 used for training and 20 used for testing. The test accuracy rate reached 90%, and the results of the testing model on the slice samples basically coincided with the opinions of medical experts.

**Fig. 4:**
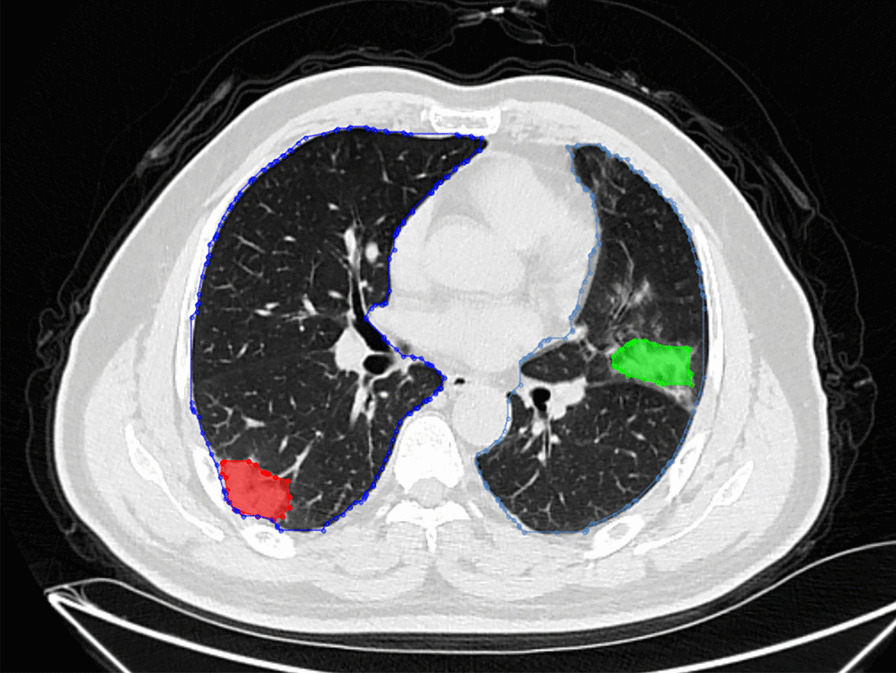
Schematic diagram of filing of patients’ CT images

**Fig. 5:**
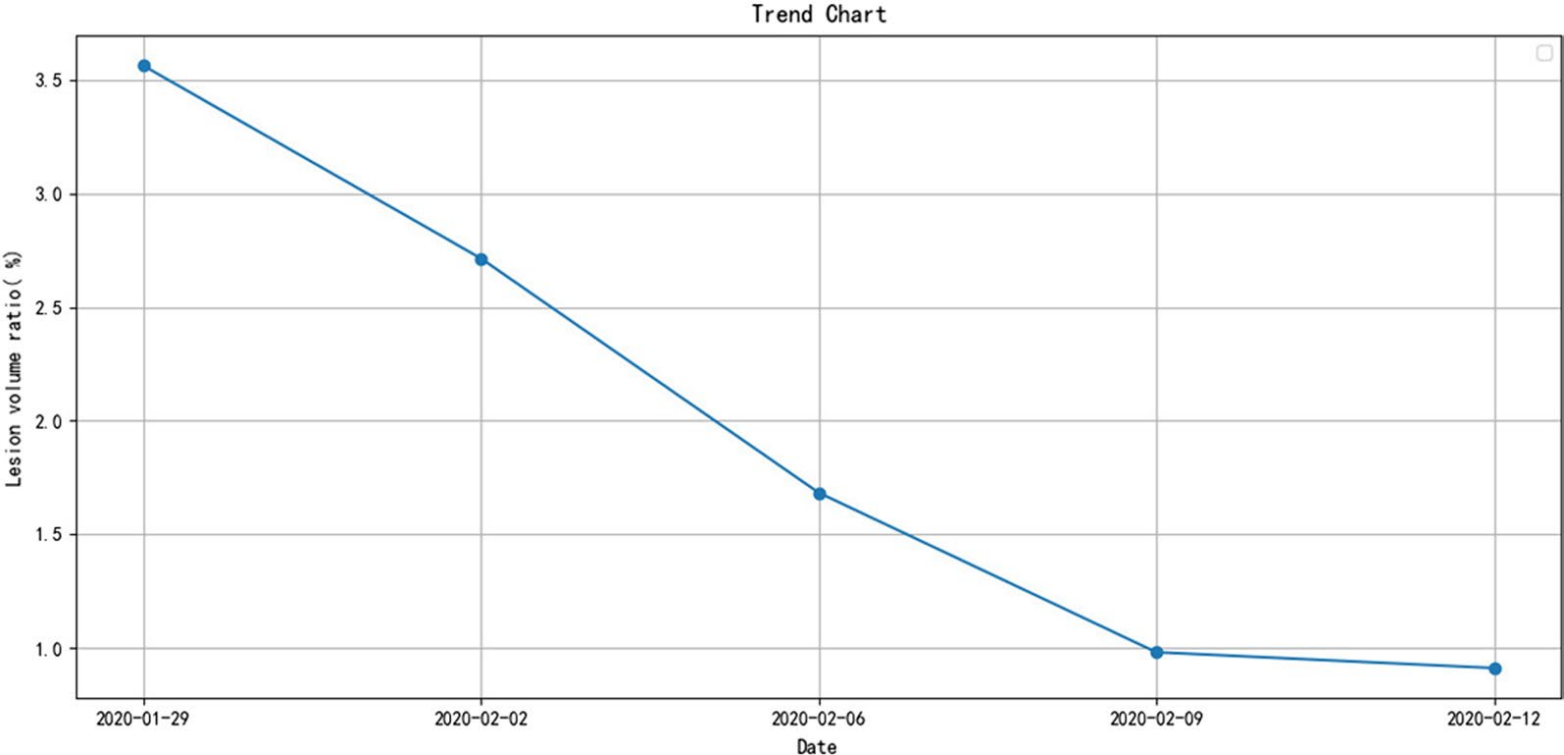
Schematic diagram of the CT lesion area of a patients’ lungs

The polygonal contours on the CT cross-section of the lungs were the focuses of infection predicted by the model (Fig. 4). Based on the deep learning network of Mask R-CNN, lung contours and the focuses of infection were extracted from CT images, and we generated 3D lesion data through intelligent matching and calculated the quantitative factors of COVID-19(Fig. 5). On the construction of the combined prediction model, 617 CT samples were utilized for testing, 522 of which were from critically ill patients, and the remaining 95 were samples from normal healthy people. On the basis of the deep neural network, we obtained the quantitative factors of the CT samples, and then performed the threshold discrimination. COVID-19 detection has an 86% sensitivity and an 85% specificity.

### In-depth integration of CT radiomics features and clinical parameters to predict the severity of COVID-19

**Fig. 6:**
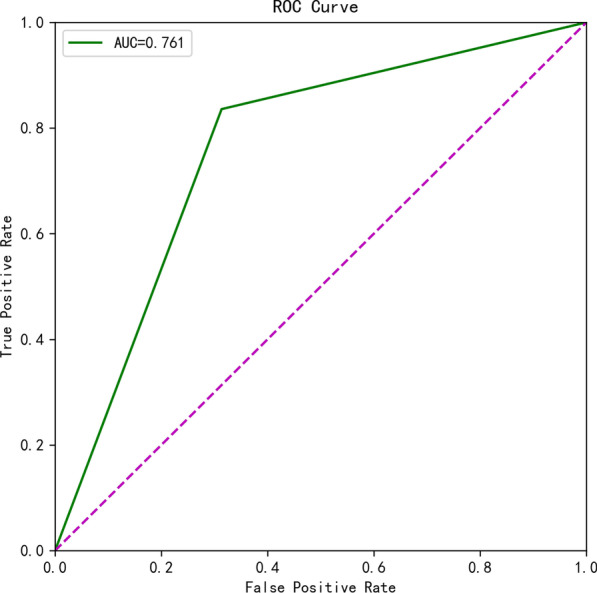
Trend graph drawn on the basis of patients’ lesion information (quantification factor) on CT images

Following that, we employed artificial neural networks to create a prediction model for the severity of COVID-19 by combining distinctive imaging features on CT and clinical parameters. Among the 205 COVID-19 patients, 140 patients with both CT imaging and clinical data were selected, with 70 critically ill and 70 mildly ill. 11 imaging features were extracted from CT samples using the deep neural network and combined with 17 clinical measurement indicators during the hospitalization of the patient. The SelectKBest method was used to select the best 15 feature combinations from 28 features (Table 2). The ANN neural network was utilized for training, and the prediction model was verified using tenfold cross-validation. As shown in Fig. 6, the area under the curve (AUC) of the prediction model is 0.761, and the sensitivity and specificity of the model are 79.1% and 73.1%, respectively, reaching a prediction accuracy of 76.1%.

**Table 2:** CT image characteristics and clinical parameters

CT image characteristics	Clinical parameters	Feature combination
Number of lesions	Leukocyte	Total lesion volume
Total lesion volume	Neutrophil count	Volume change
Mean lesion volume	Neutrophil ratio	Proportion of lesions
Proportion of lesions	Lymphocyte count	Mean density
Maximum 3D long diameter	Lymphocyte ratio	Edge clarity
Maximum 2D long diameter	Monocyte count	Pleural distance
Mean density	Proportion of monocytes	Form
Edge clarity	Eosinophil count	Mean lesion volume
Pleural distance	Eosinophil ratio	Neutrophil ratio
Form	Red blood cell count	Lymphocyte ratio
Lesion volume change	Hemoglobin	Red blood cell count
	Hematocrit	Hematocrit
	Platelet count	Platelet count
	Alanine aminotransferase	Alanine aminotransferase
	Aspartate aminotransferase	PaO2
	Albumin	
	PaO2

### Building the AI lung image recognition processing technological platform on the basis of the constructed deep neural network model

**Fig. 7:**
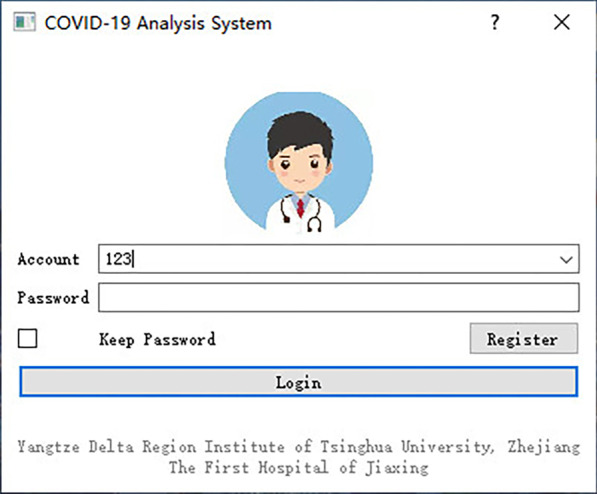
Visualization platform for CT image features

The research takes AI imaging technology as the core; CT imaging sample data of patients with COVID-19 as a motivator; servers, databases, and human–computer interaction interfaces as carriers to build an AI imaging platform with practical value. Relevant medical workers can log into the platform (Fig. 7) and use the functions with corresponding permissions. In the later stage, the account authority can be shared with the existing system of the hospital to realize the integration of the system platform.

**Fig. 8:**
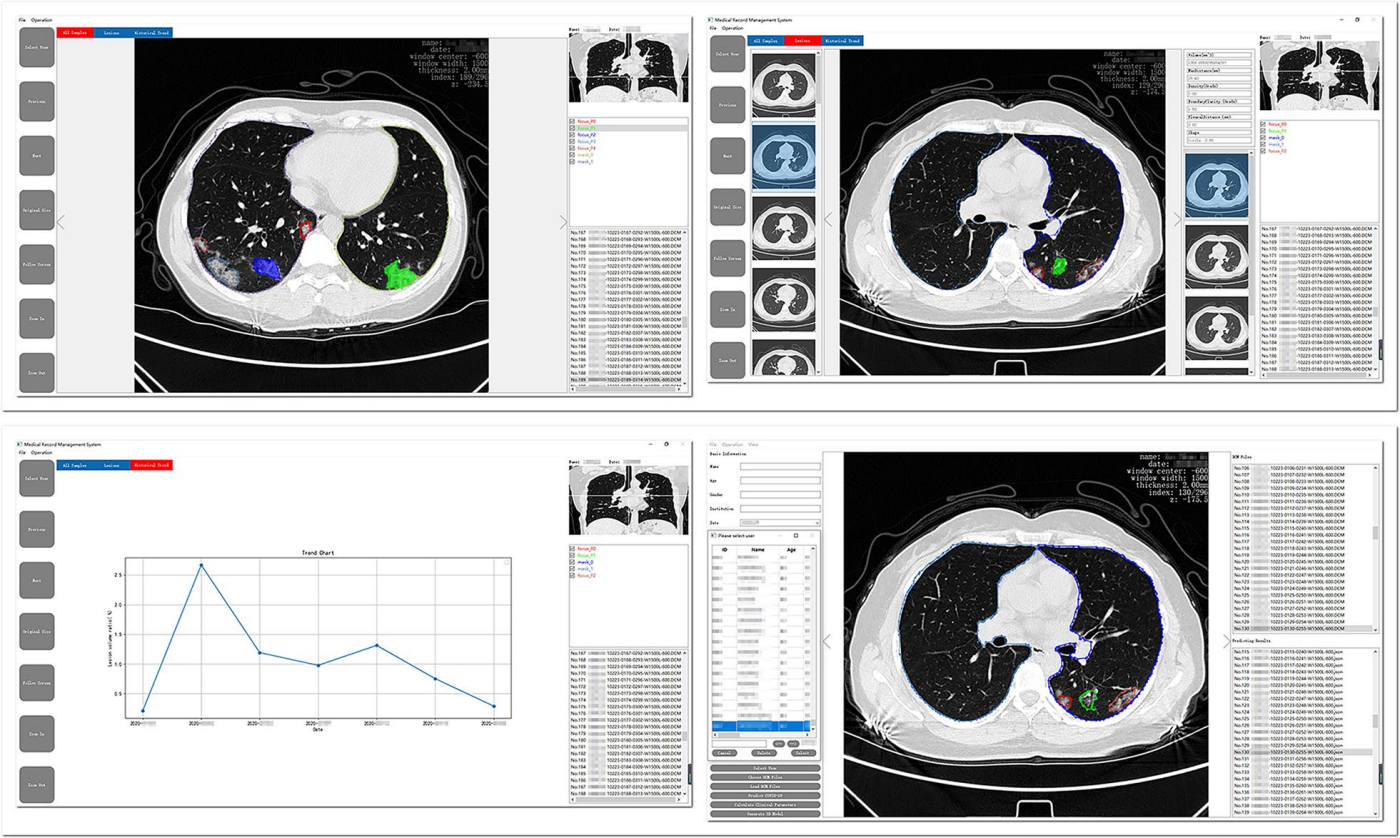
3D images of lesions. The 6 quantitative factors are as follows: i Number of lesions: calculating the number of lesions by 3D lesion data. The more the lesions, the more severe the condition. ii Maximum lesion length: calculating the maximum lesion length of each lesion. The larger the lesion, the more severe the condition. iii Lesion density: Generally, the more uneven the density of the lesions, the more severe the disease, and the higher the density, the more serious the disease. iv Lesion boundary: usually the boundaries of inflammation are blurry, and the boundaries of diseases such as cancer are clearer. v Distance from the edge of the lesion to the pleura: One of the characteristics of COVID-19 is that it often occurs under the pleura. vi The shape of the largest 3 lesions: usually the safer lesions are regular in shape, that is, round or oval, and the dangerous lesions are irregular in shape

**Fig. 9:**
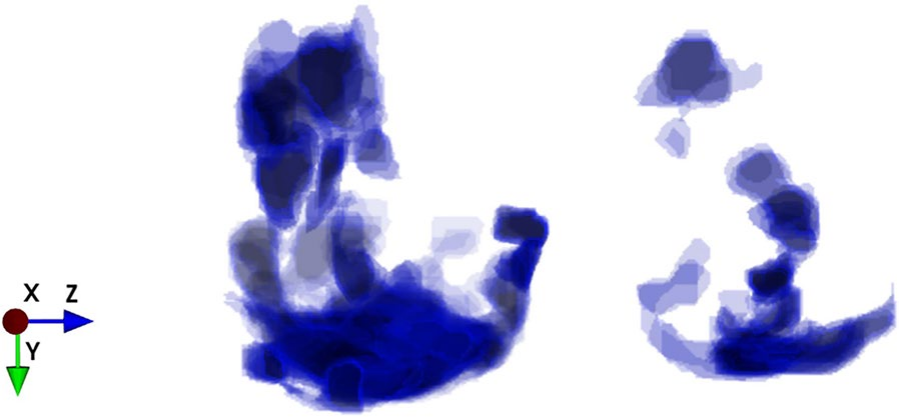
ROC curve of feature combination model

The platform can display lesion images, parameters, variation tendency of the disease, etc. (Fig. 8). The lesion information and severity of the pneumonia collected from the samples using the aforementioned AI model will be saved in the COVID-19 AI technology platform.Then, we can combine medical experience with calculated quantitative factors (Fig. 9) to explain the severity of the disease.

## Discussion

COVID-19 is an acute contagious disease with a high transmission rate and spreading rapidity, which has caused a global pandemic [4]. Chest CT is an important standard for diagnosis and discharge, and it plays a important role in the diagnosis, disease evaluation, and efficacy evaluation of COVID-19 [12]. However, CT may have certain imaging features in common between COVID-19 and other types of pneumonia, making differentiation difficult [27]. AI technology represented by deep learning has made a breakthrough in the domain of medical imaging [28, 29]. The image learning method, segmentation and applications in lung diseases are the research hotspots of AI in medical imaging with high clinical application potential [30]. Deep learning has been applied to detect and differentiate between bacterial and viral pneumonia on pediatric chest radiographs [31]. In this study, we proposed to build a severe COVID-19 early warning model based on the deep learning network of Mask R-CNN and chest CT images and patient clinical characteristics. We hope to make early predictions of severe COVID-19 patients by this model.

In recent years, an artificial intelligence imaging diagnosis system that can perform quantitative analysis and differential diagnosis of lung inflammation has become a research hotspot [16]. AI technology can extract image data information quickly and in parallel, allowing for a more comprehensive and detailed analysis of the nature of the lesion from the aspects of overall characteristics, peripheral characteristics, internal characteristics, and surrounding tissues, as well as other clinical characteristics of patients [21, 32]. The radiologic diagnostic tool built by AI technology for the diagnosis of COVID-19 has been confirmed to be helpful for the early screening of COVID-19 pneumonia [33, 34]. Li L et al. developed an AI program based on the results of chest CT scans. The sensitivity and specificity of the program for diagnosing patients with COVID-19 pneumonia were 90% and 96%, respectively [35]. Shi et al. used data from 1,658 COVID-19 patients and 1,027 community-acquired pneumonia patients to generate an AI program, and used five-fold cross-validation to obtain 90% sensitivity and 83% specificity in the detection of COVID-19 pneumonia [36]. On the other hand, Shan et al. created an AI program to assess the extent of lesion spread using the V-net and V-bet-based networks, and its Dice similarity coefficient was 91.6 ± 10(%), with an estimated error of the percentage of infection (POI) of 0.3 percent [37]. In this research, we used the Mask R-CNN deep neural network model to extract lung contours and lesion locations from CT images to generate 3D lesion data, and to calculate quantification factors for COVID-19 [38]. The quantification parameters of CT samples obtained using the deep learning network showed a sensitivity of 96% and a specificity of 85% for detecting COVID-19. Additionally, we combined CT image characteristics with clinical parameters and applied an AI neural network to develop a prediction model for the severity of COVID-19. The model, which was validated using tenfold cross-validation, had an AUC of 0.761 for detecting patients with severe COVID-19, and the sensitivity and specificity were 79.1% and 73.1%, respectively, showing that the model performed effectively.

This research builds an early warning model for severe COVID-19, which has a certain innovative contribution. Firstly, it overcomes the bottleneck of predictingwhether patients will become critically ill in the diagnosis and treatment of COVID-19 [39], and build a highly accurate early warning model for COVID-19, which can help health workers hierarchically manage confirmed patients and intervene in the diagnosis and treatment of high-risk patients in advance to improve patient prognosis and reduce social medical costs at the same time. In addition, the image features extracted by traditional radiomics methods are low-level or intermediate-level features, and these functions are not detailed enough to illustrate the deep information of the images. This research uses deep learning to extract the advanced radiological characteristics of CT images, and combines traditional radiomics and key clinical data to construct a high-performance early prediction model to realize the early warning of severe COVID-19. Furthermore, deep learning can provide more effective imaging features than conventional radiomics, but its main limitation, the black box, restricts its clinical application and promotion. This research uses the attention mechanism to assess the importance of each potential feature or component learned by the model in order to increase the accuracy of early warning of severe COVID-19 and to visualize and interpret these important features using statistical analysis of clinical data.Visualization and interpretability of the clinical data will help clinicians in developing principles and criteria for the hierarchical diagnosis of COVID-19 patients based on an early warning model.

However, there are certain limitations to this study. The small sample size is one of the most significant limitations. Although the results of utilizing AI models to diagnose and predict whether COVID-19 patients will become severe are encouraging, more data is needed to validate the model's universality. Moreover, the model's training and verification are limited to a small number of domestic populations, and we hope that international populations can be employed to further validate and increase the model's universality. It is important to note that the AI image recognition platform built by this research is extensible.The AI image recognition platform can not only seamlessly include other lung diseases into the platform, but also other diseases based on image recognition, such as the identification of restricted airways (Fig. 1). We hope that the system can be developed into a multi-functional tool against COVID-19 and other emerging virus infections.

## Conclusions

In conclusion, based on deep learning, the combined prediction model for severe COVID-19 pneumonia has a strong differential ability for predicting the course of disease in COVID-19 patients by combining clinical features, pulmonary lesion volume, and radiomics features. This may assist in the early prevention of severe COVID-19 symptoms.

## Data Availability

The data supporting this article are available from the corresponding author on reasonable request.

## Abbreviations

RNN: Recurrent neural network
CL: Compliance of lung
ANN: Artificial neural network
ROC: Receiver operating characteristic
SARS-CoV-2: Severe acute respiratory syndrome coronavirus 2
ICU: Intensive care unit
WHO: World Health Organization
RT-PCR: Reverse transcription-polymerase chain reaction
CT: Computed tomography
AUC: Area under the curve
